# Supplementary material for: A retinoraphe projection regulates serotonergic activity and looming-evoked defensive behaviour
Source: Nat Commun. 2017 Mar 31;8:14908. doi: 10.1038/ncomms14908 (PMC5381010; doi:10.1038/ncomms14908)
Supplement: Supplementary Information — Supplementary Figures. [file ncomms14908-s1.pdf]

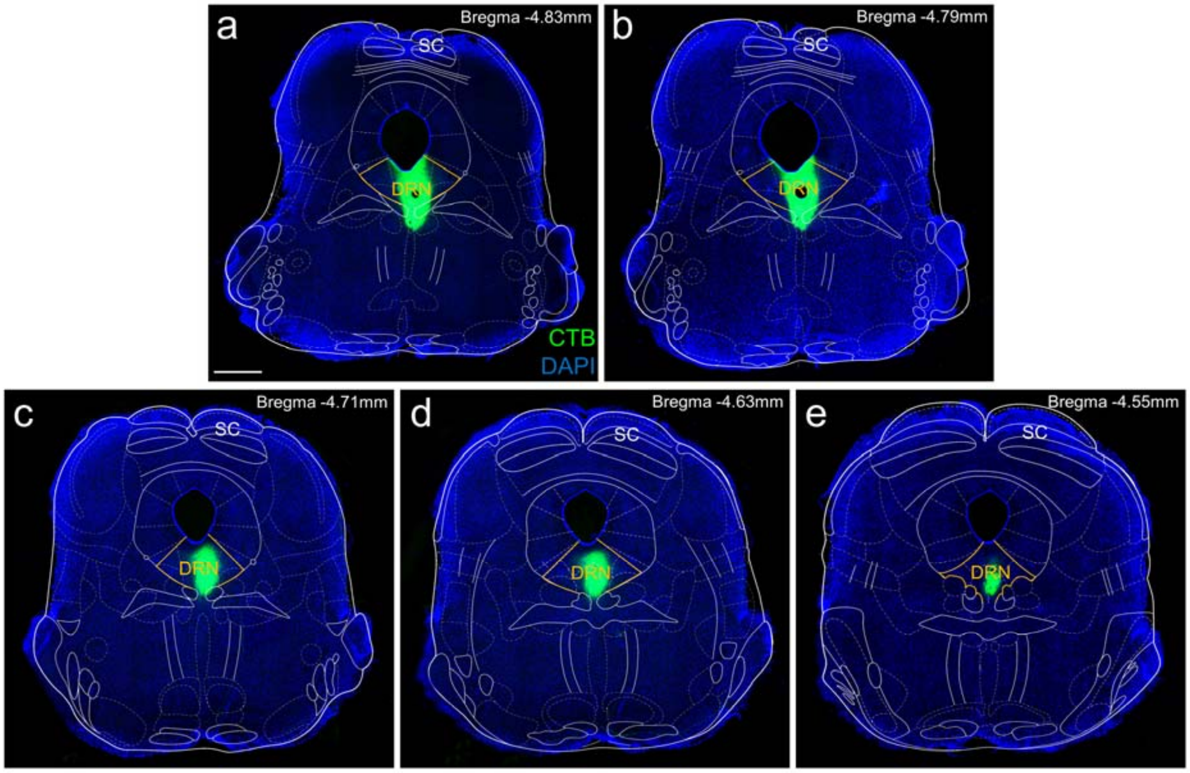

Supplementary Figure 1 CTB-488 DRN injection site which avoided the SC. (**a**-**e**) A representative example of CTB-488 deposited into the DRN of a C57BL/6 mouse illustrated in a series of 40 μm sections from caudal to rostral. Note SC is unlabeled. Scale bar: (**a**) 1 mm.

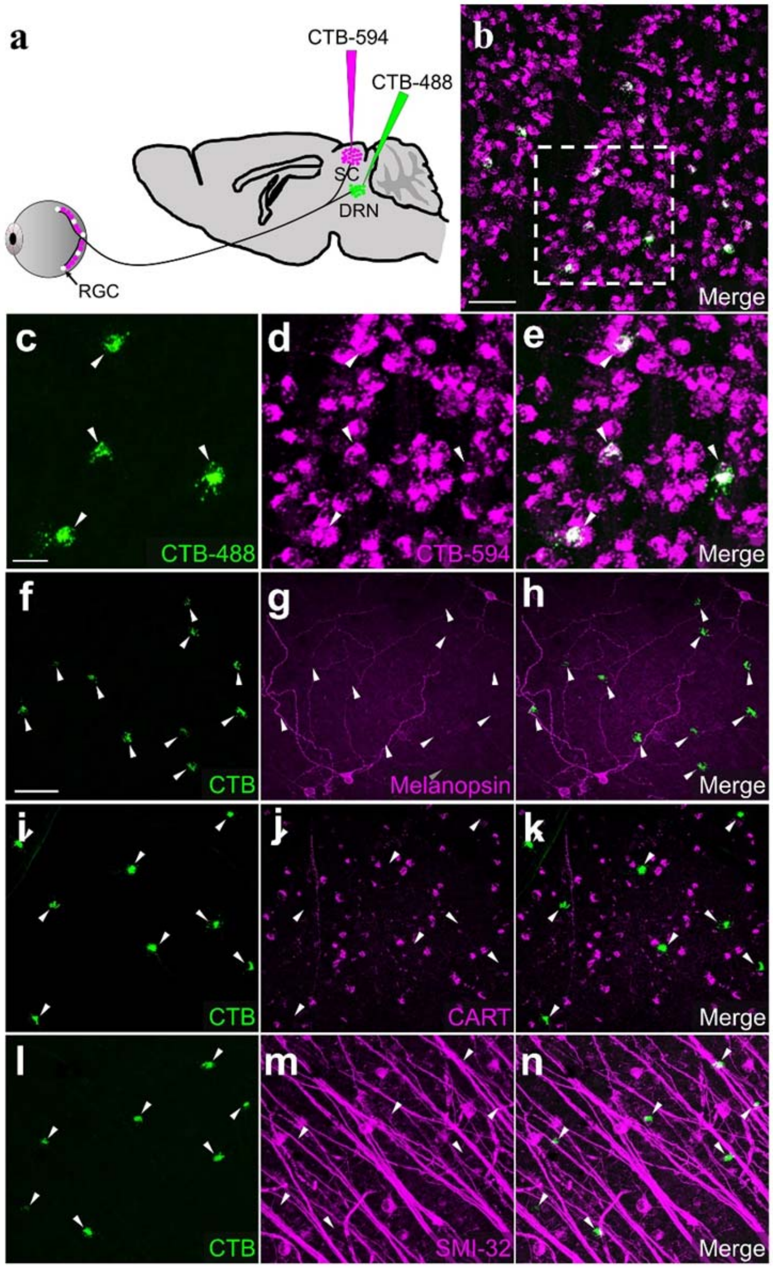

Supplementary Figure 2 DRN-projecting RGCs innervate the DRN and SC via branching axons and these neurons are immunonegative for melanopsin, CART, and SMI-32. (**a**) Injection paradigm used to label RGCs with branching axons innervating the DRN and SC. (**b**) A merged image of a retinal whole mount illustrating double-labeled RGCs that innervate the DRN and SC. (**c-e**) Region defined in (**b**) viewed under higher magnification illustrating CTB-488 labeled DRN-projecting RGCs (arrow heads) (**c**) and CTB-594 labeled SC-projecting RGCs (**d**), and merged image (**e**). (**f-n**) Retinal whole mounts containing CTB-labeled DRN-projecting RGCs that were subjected to immunostaining for melanopsin, CART, and SMI-32 and no doubled-labeled RGCs were observed. Scale bars: (**b** and **f**) 50 μm; (**c**) 20 μm.

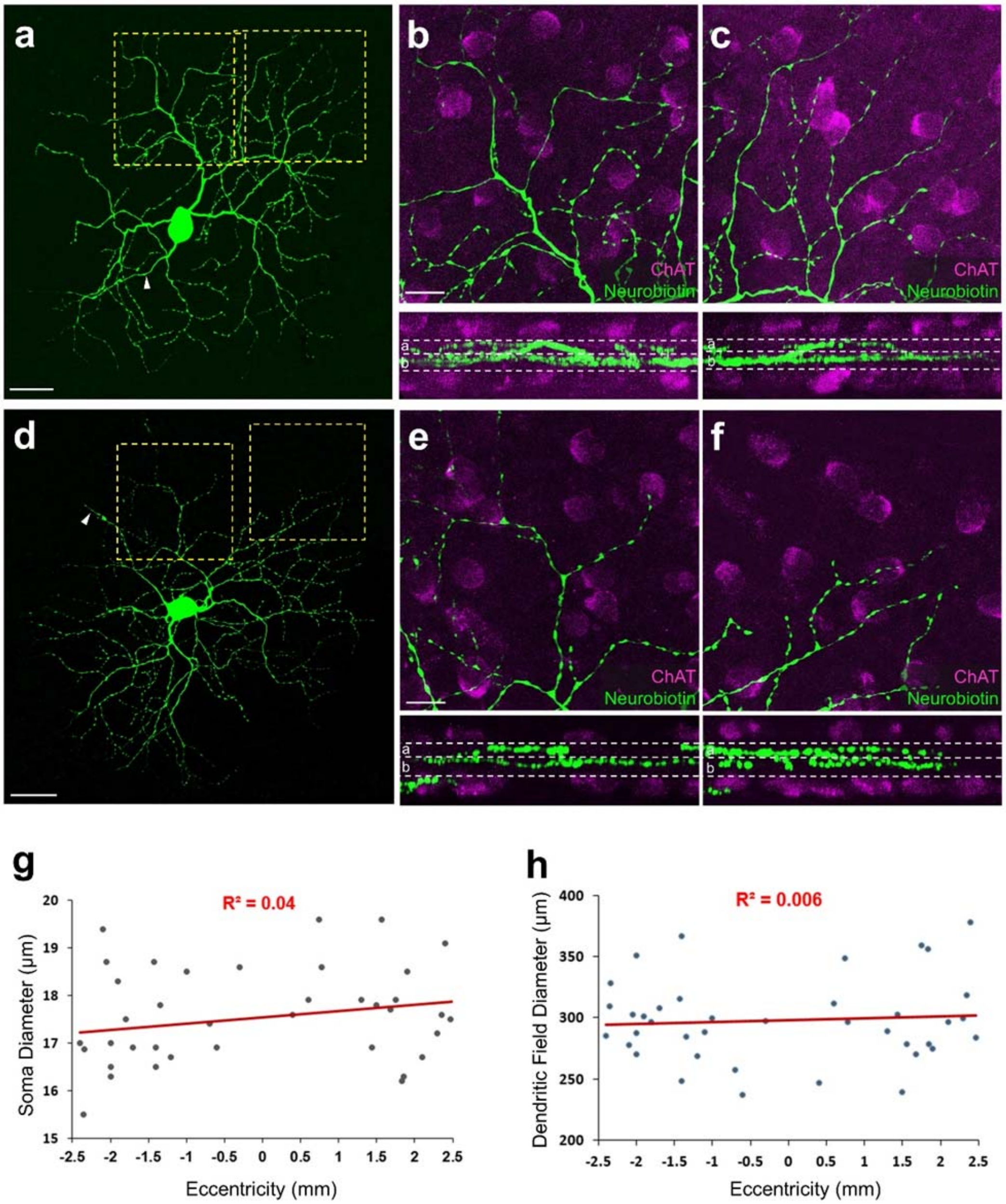

Supplementary Figure 3 Dendritic morphology of DRN-projecting RGCs. (**a-f**) Two DRN-projecting RGCs filled with neurobiotin. Note that they both have an asymmetric dendritic field, and bistratified dendritic arbors. (**g,h**) Plot of soma diameter (**g**) and dendritic field diameter (**h**) as a function of retinal eccentricity. 3 animals were used in each analysis. Scale bars: (**a** and **d**) 20 μm; (**b** and **e**) 10 μm.

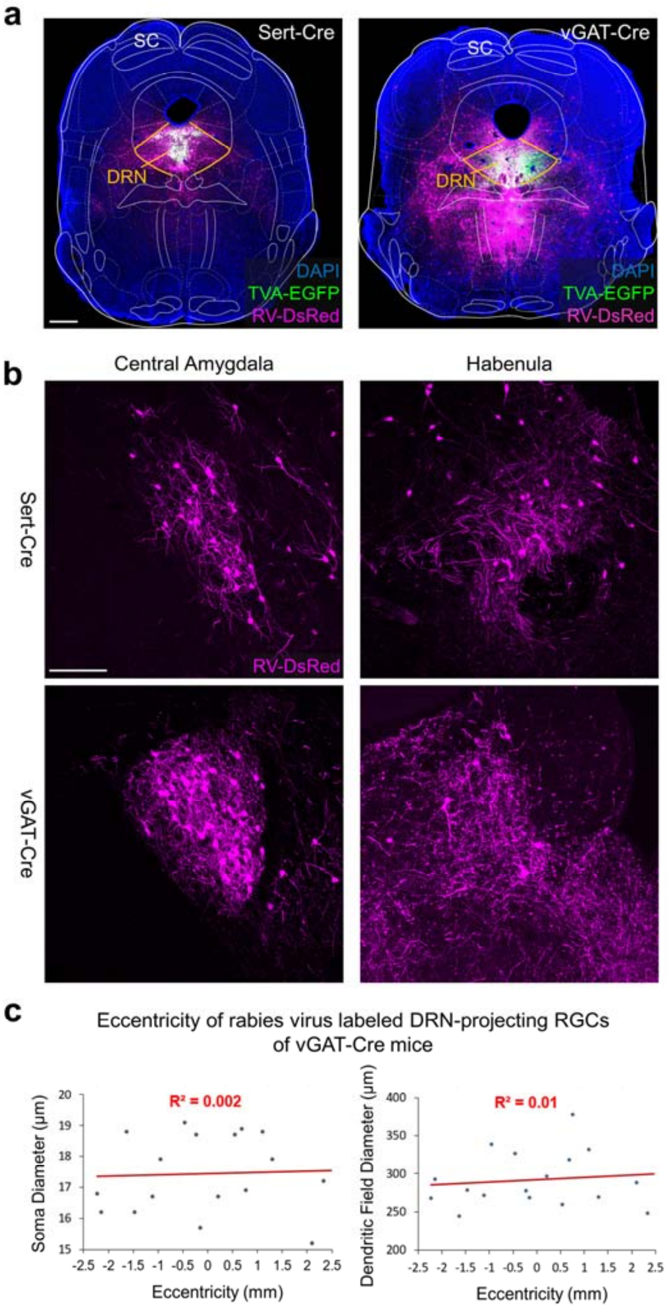

Supplementary Figure 4 Rabies virus labeled monosynaptic inputs from central amygdala and habenula to DRN 5-HT and GABA neurons. (**a**) Coronal sections of a Sert-Cre (left) and a vGAT-Cre (right) brain showing injection sites of rabies-based transsynaptic system. Note that the location of the starter cells (white) is confined to the DRN. (**b**) Coronal sections through the central amygdala and habenula from a Sert-Cre mouse and a vGAT-Cre mouse showing the distribution of presynaptic neurons (magenta). (**c**) Plot of soma diameter and dendritic field diameter as a function of retinal eccentricity of rabies-labeled DRN-projecting RGCs. Scale bars: (**a**) 500 µm; (**b**) 100 µm.

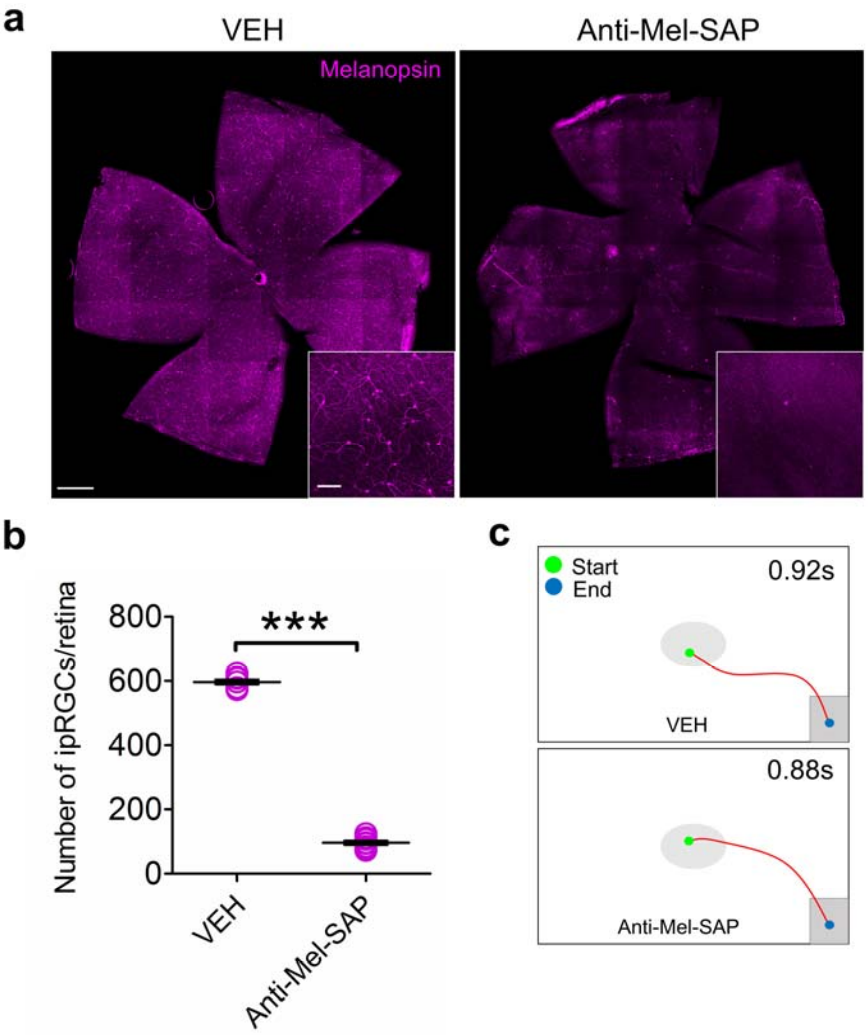

Supplementary Figure 5 Selective immunotoxin ablation of melanopsin-expressing RGCs does not influence looming induced defensive response. (**a**) Retinal whole mounts illustrating melanopsin-expressing RGCs in animals receiving intraocular injections of saline (VEH) or anti-melanopsin-saporin immunotoxin (Anti-Mel-SAP). (**b**) Quantification showing Anti-Mel-SAP reduction of melanopsin-expressing RGCs. One-way ANOVA; *** $p<0.0001$. (**c**) Representative traces of animal movement during looming stimulation (10.75 s) in VEH and Anti-Mel-SAP groups. Scale bars: (**a**)-left 500 μm; (**a**)-inset 100 μm. Data represented as mean ± s.e.m.

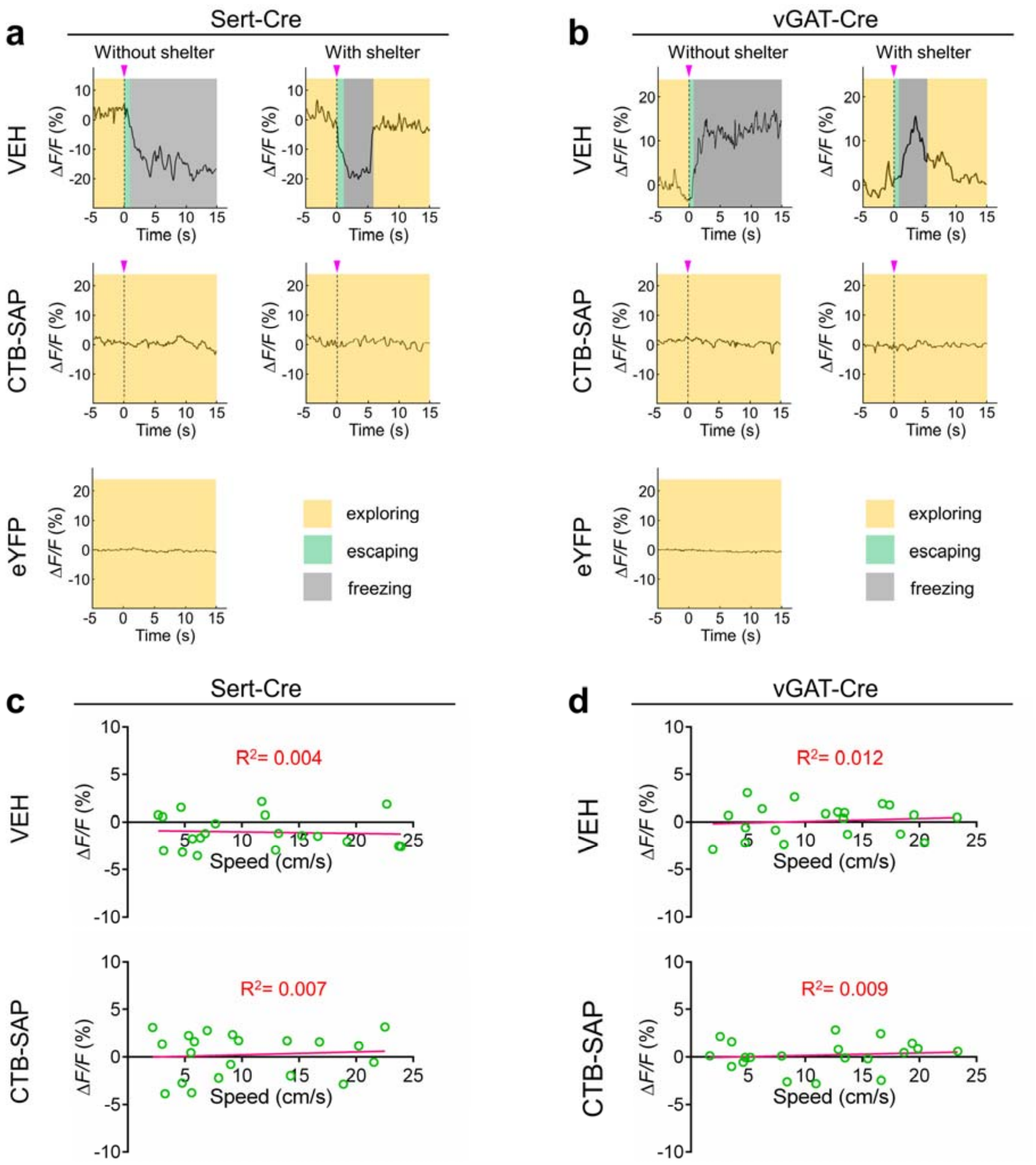

Supplementary Figure 6 Relationship between behavior and GCaMP fluorescence changes during looming stimulation. (**a,b**) VEH: Representative raw traces of GCaMP fluorescence changes in response to looming stimulation in VEH-treated Sert-Cre/vGAT-Cre mice; CTB-SAP: Representative raw traces of GCaMP fluorescence changes in response to looming stimulation in anti-CTB-SAP treated Sert-Cre/vGAT-Cre mice. eYFP: measurement of potential movement artifact from DRN 5-HT or GABA neurons expressing eYFP. (**c**) Correlation between speed and GCaMP fluorescence changes in VEH or anti-CTB-SAP treated Sert-Cre mice. (**d**) Correlation between speed and GCaMP fluorescence changes in VEH or anti-CTB-SAP treated vGAT-Cre mice.

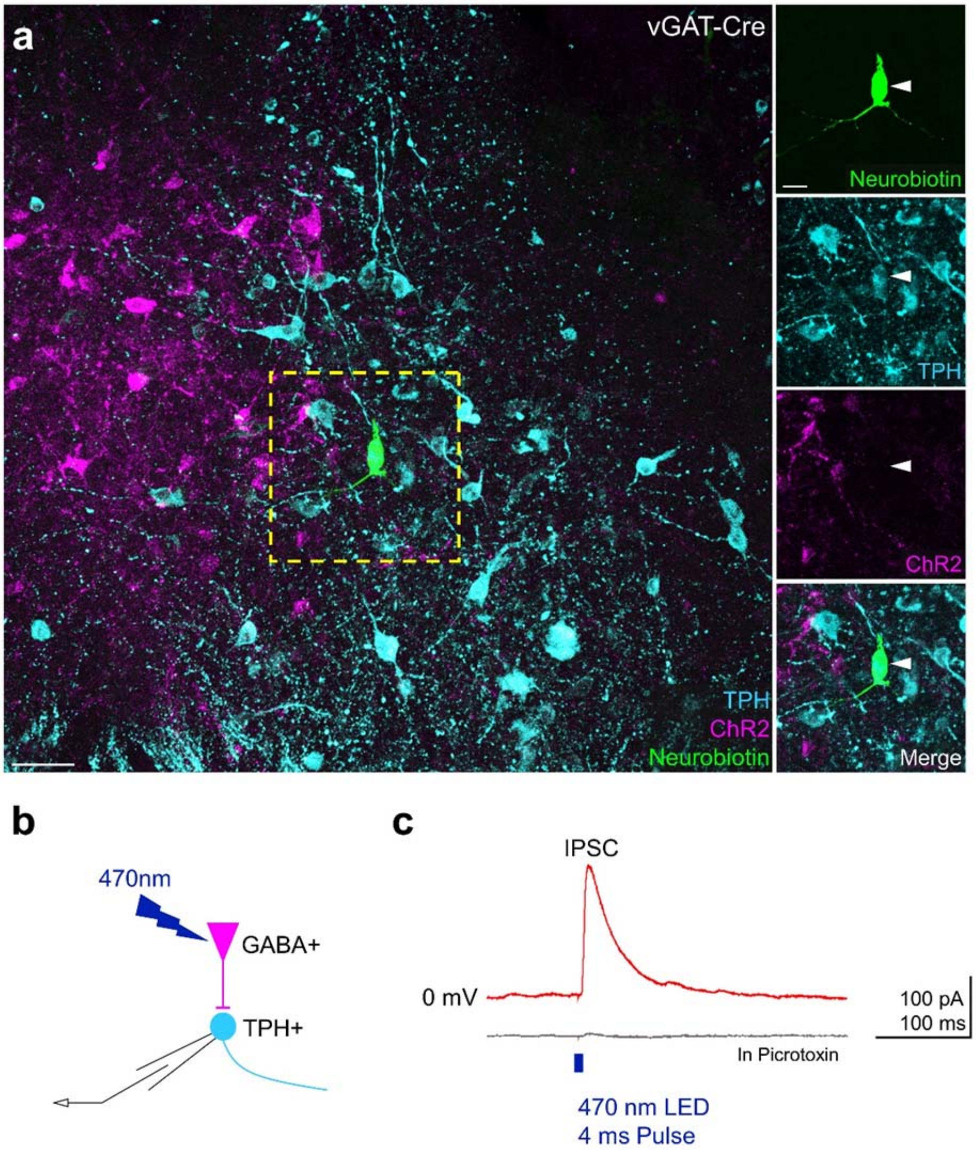

Supplementary Figure 7 Optogenetic activation of DRN GABA neurons *in vitro* inhibits DRN 5-HT neurons. (**a**) Merged image illustrating a neurobiotin-filled 5-HT neuron in a DRN slice from a vGAT-Cre mouse in which GABA neurons were transfected with AAV-DIO-ChR2-mCherry. (**b**) Before neurobiotin filling of the 5-HT neuron illustrated in (**a**) it was recorded using the whole-cell patch-clamp technique while the slice was stimulated with blue (470 nm) light. (**c**) The recorded 5-HT neuron was inhibited by optogenetic activation of DRN GABA neurons via a $GABA_A$ receptor-mediated mechanism as the evoked-IPSC was blocked by bath application of 100 µM picrotoxin. Scale bars: (**a**)-left 50 µm; (**a**)-right 20 µm.

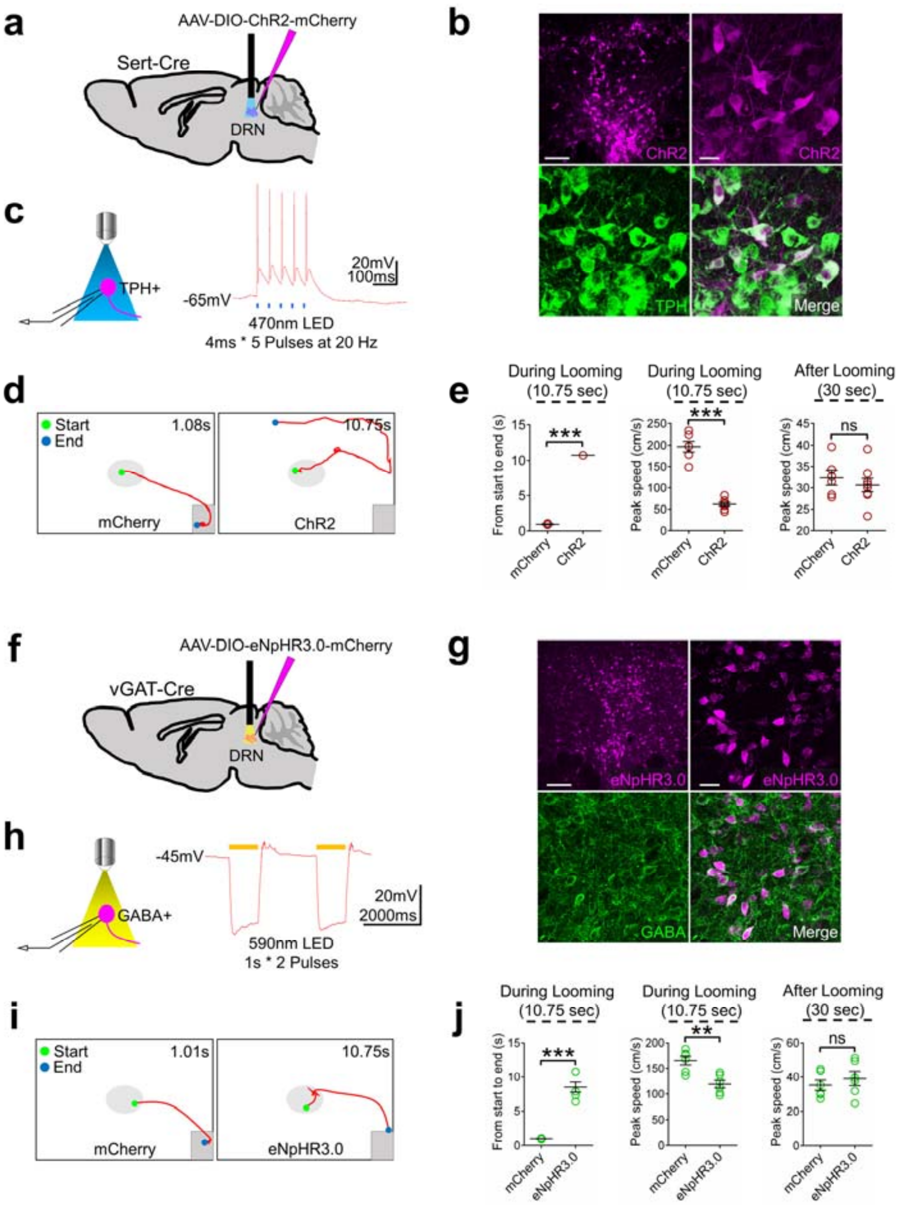

Supplementary Figure 8 Optogenetic activation of DRN 5-HT neurons inhibits looming-evoked response. (**a**) AAV-DIO-ChR2-mCherry was injected into DRN of Sert-Cre mice. (**b**) Images of TPH cells in DRN of a Sert-Cre mouse two weeks after ChR2 transfection. (**c**) Representative example of optogenetic (470nm blue light) activation of a DRN 5-HT neuron in an in vitro slice preparation. (**d**) Representative traces of animal movement when DRN 5-HT neurons were optogenetically activated during looming stimulation (10.75 sec). Time in corner is duration from start to end. (**e**) Duration from start to end points, peak speed during and after looming stimulation in mCherry and ChR2 treated animals. One-way ANOVA; *** $p<0.0001$; ns = no significant difference. (**f**) AAV-DIO-eNpHR3.0-mCherry was injected into DRN of vGAT-Cre mice. (**g**) Images of GABA cells in DRN of a vGAT-Cre mouse two weeks after eNpHR3.0 transfection. (**h**) Representative example of optogenetic (590nm yellow light) inhibition of a DRN GABA neuron in an in vitro slice preparation. (**i**) Representative traces of animal movement when DRN GABA neurons were optogenetically inhibited during looming stimulation (10.75 sec). Time in corner is duration from start to end. (**j**) Duration from start to end points, peak speed during and after looming stimulation in mCherry and eNpHR3.0 treated animals. One-way ANOVA; *** $p<0.0001$; ** $p<0.001$; ns = no significant difference. Scale bars: (**b** and **g**)-left 100 µm; (**b** and **g**)-right 20 µm. Data represented as mean ± s.e.m.
